# IGF2BP2 serves as a core m6A regulator in head and neck squamous cell carcinoma

**DOI:** 10.1042/BSR20221311

**Published:** 2022-11-11

**Authors:** Yuan Hu, Jiexin Chen, Muyuan Liu, Qin Feng, Hanwei Peng

**Affiliations:** 1 Department of Head and Neck Surgery, Cancer Hospital of Shantou University Medical College, Guangdong 515041, P.R. China; 2 Department of Rheumatology, First Affiliated Hospital of Shantou University Medical College, Guangdong 515041, P.R. China

**Keywords:** Clinical significance, Methylation of N6 adenosine, Squamous Cell Carcinoma of Head and Neck

## Abstract

Methylation of N6 adenosine (m6A) plays a crucial role in the development and progression of cancers. Its modification is regulated by three types of m6A-related regulators (methyltransferases (writers), demethylases (erasers), and RNA-binding proteins (readers)). Till now, the functions and roles of these regulators in head and neck squamous cell carcinoma (HNSC) remain largely unexplored. Therefore, we utilized the open HNSC dataset in The Cancer Genome Atlas (TCGA), four different cell lines, and our HNSC patient samples (*n*=40) to explore the clinical significance of 19 m6A regulators, and selected the most significant prognosis-related regulator. Authentic analyses based on online websites were also used in the study (Oncomine, UALCAN, Kaplan–Meier plotter, Human Protein Atlas (HPA), cBioPortal, LinkedOmics, String, etc.). From the results, general overexpression of m6A regulators was observed in pan-cancer, especially in HNSC. IGF2BP2 was recognized as the hub m6A regulator, which was an independent, unfavorable prognostic factor in HNSC. Its mRNA and protein expression in HNSC were significantly up-regulated. Gene mutation types of IGF2BP2 in HNSC (32%) were mainly mRNA High or Amplification, which represented the high expression of IGF2BP2. And these mutations were associated with a poor prognosis. In functional analysis, IGF2BP2 was negatively correlated to tumor immune infiltration in HNSC. Finally, HMGA2 might interact with the IGF2BP2 in HNSC. In conclusion, IGF2BP2 serves as a core m6A regulator among all regulators in HNSC, which has a high expression and predicts the poor prognosis of HNSC patients independently. IGF2BP2 might bring a new direction for HNSC treatment in the future.

## Introduction

Head and neck squamous cell carcinoma (HNSC) is currently the sixth most common cancer in the world. Approximately 65 thousand new cases and 14 thousand deaths due to HNSC occurred in the United States in 2019 [1]. HNSC can cause many harms to the body and is lethal, such as cosmetic deformity and functional impairment of vital functions, including breathing, swallowing, phonating, hearing, smelling, etc. [2]. The treatment for early-stage HNSC is either surgery or radiotherapy, while for locally advanced HNSC is multimodal, with either surgery followed by adjuvant radiation or chemoradiation [3–5]. Despite our best efforts, the overall survival (OS) of HNSC patients has not been improved significantly during the past decades. The 5-year OS rate of locally advanced patients is only 50%, and the OS of patients with recurrence or metastasis is about 6 months [2,6,7]. Therefore, there is a dire need to find new biomarkers for early screening and effective treatment of HNSC.

Methylation of N6 adenosine (m6A), a prevalent type of RNA modification, might be a new direction for improving the prognosis of HNSC. M6A plays a crucial role in the development and progression of cancers [8,9]. It can participate in the tumorigenesis and metastasis of malignancies by regulating the expression of cancer-related genes through splicing, processing, translation, and decay [10]. Its modification is regulated by three types of regulators, including methyltransferases (writers), demethylases (erasers), and RNA binding proteins (readers) [8]. The writers (METTL3, METTL14, WTAP, RBM15, RBM15B, ZC3H13) can catalyze the formation of m6A on RNA; the erasers (FTO, ALKBH5) can help to remove the methyl modification from target RNAs; the readers (YTHDF1, YTHDF2, YTHDF3, YTHDC1, YTHDC2, HNRNPA2B1, IGF2BP1, IGF2BP2, IGF2BP3, RBMX, HNRNPC) can identify specific m6A modification and produce a functional signal [8–10]. Till now, many studies have proven that these regulators are significantly associated with cancers [11]. It was found that METTL3 and METTL14 (writers) served an oncogenic role in acute myeloid leukemia (AML) in an m6A manner by promoting the translation of MYC, MYB, BCL2, SP1, and PTEN [12,13]. Several studies showed that FTO and ALKBH5 (erasers) expression were associated with a less favorable prognosis in patients with glioblastoma (GBM) [14,15]. In addition, YTHDC2 and IGF2BPs (readers) might lead to metastasis of colorectal cancer (CRC) by up-regulating HIF-1α or c-Myc expression through m6A modification [16,17].

So far, only a few researches have been conducted on the functions of m6A regulators in HNSC. Among them, METTL3 is studied the most, which showed a cancer-promoting role in HNSC. It was found that METTL3 promoted tumorigenesis and metastasis through promoting BMI1 m6A methylation in oral squamous cell carcinoma [18]. As more and more m6A regulators are discovered, the relationship between each m6A regulator and HNSC remains largely unexplored. In the present study, we enrolled known m6A regulators to systematically study their clinical value in HNSC, aiming to identify the hub m6A regulators in HNSC based on online data and our own data.

## Materials and methods

### m6A Regulators

A total of 19 m6A regulators were studied in the study: six writers (methyltransferase like 3, METTL3; methyltransferase like 14, METTL14; WT1-associated protein, WTAP; RNA-binding motif protein 15, RBM15; RNA-binding motif protein 15B RBM15B; zinc finger CCCH-type containing 13, ZC3H13), two erasers (FTO α-ketoglutarate-dependent dioxygenase, FTO; alkB homolog 5, RNA demethylase, ALKBH5), 11 readers (YTH domain-containing 1, YTHDC1; YTH domain-containing 2, YTHDC2; YTH N6-methyladenosine RNA-binding protein 1, YTHDF1; YTH N6-methyladenosine RNA-binding protein 2, YTHDF2; YTH N6-methyladenosine RNA-binding protein 3, YTHDF3; insulin-like growth factor 2 mRNA-binding protein 1, IGF2BP1; insulin-like growth factor 2 mRNA-binding protein 2, IGF2BP2; insulin-like growth factor 2 mRNA-binding protein 3, IGF2BP3; RNA-binding motif protein X-linked, RBMX; heterogeneous nuclear ribonucleoprotein C, HNRNPC).

### mRNA expression analysis in Oncomine

The transcriptional levels of 19 m6A regulators between different cancer tissues and their corresponding adjacent normal controls were identified in Oncomine (https://www.oncomine.org) [19]. The differences were compared by *t*-test (*P*=0.01; fold-change, 1.5; gene rank, 10%; data type, mRNA).

### mRNA expression analysis in UALCAN

The mRNA expressions of 19 m6A regulators between HNSC tissues and normal controls were analyzed by UALCAN (http://ualcan.path.uab.edu) [20]. For the hub m6A regulators, we performed the stratified analysis based on patients’ age, gender, individual cancer stage, and human papillomavirus (HPV) status. The differences in transcriptional level were compared by *t*-test, and *P*<0.05 was considered statistically significant. The m6A regulators that had no statistical differences in expression were excluded for further analyses.

### Survival analysis in Kaplan–Meier plotter

The prognostic value of the selected m6A regulators in HNSC was analyzed in Kaplan–Meier plotter (http://kmplot.com/) [21]. The prognosis-related m6A regulators were chosen for further research. It was considered statistically significant when Cox *P-*value<0.05 in Kaplan–Meier plotter.

### Statistical methods

Transcriptome data of HNSC from The Cancer Genome Atlas (TCGA) was used to conduct further analyses, which was downloaded from the UCSC Xena. To verify the prognostic value of the selected m6A regulators, we used these data to conduct univariate and multivariate Cox regression analyses with clinicopathologic parameters. By excluding duplicate samples, we eventually included 502 HNSC patients in our study (Supplementary Table S1). The correlation analysis of gene expression was performed to predict the possible targets of the hub m6A regulator. The correlation coefficient value *r* > 0.6 is considered significant. In addition, immune checkpoint correlation analysis and tumor mutational burden (TMB) correlate analysis were also performed by using TCGA data [22]. SIGLEC15, IDO1, CD274, HAVCR2, PDCD1, CTLA4, LAG3, and PDCD1LG2 are the transcripts, which associated with the immune checkpoint. Extracting the expression of eight genes, observing the expression value of the immune-checkpoint-related genes. TMB is derived from the article *The Immune Landscape of Cancer* published by Thorsson et al. in 2018 [22]. All analyses were performed using R software (version 4.0.0). *P*<0.05 was considered statistically significant.

### Protein expression analysis and subcellular location analysis in human protein atlas

The protein expression of the selected m6A regulators between human normal and HNSC tissues was compared by using the Human Protein Atlas (HPA) (https://www.proteinatlas.org), which contains immunohistochemistry (IHC)-based expression data for near 20 highly common kinds of cancers [23]. In addition, subcellular location analysis was also explored in HPA, which adopted indirect immunofluorescence microscopy to generate images.

### IHC

To verify the protein expression difference of the hub m6A regulator, a total of 40 paired HNSC tissue specimens collected for IHC were obtained from Cancer Hospital of Shantou University Medical College, and all informed consents were obtained. The Ethical Committee of the Hospital approved the study protocol. The IHC analysis was performed using UltraSensitive SP IHC Kit-9710 (MXB, China), according to the manual instructions. The primary antibody used in IHC included: IGF2BP2 (1:250, CST). The staining intensity was scored as (0 = negative, 1 = weak, 2 = moderate, 3 = strong) according to the expression level and the staining area was scored as (0 = 0%, 1 = 1–25%, 2 = 26–50%, 3 = 51–100%) based on the percentage of positive cells. The immunostaining score (IS) was calculated by adding the score of staining intensity and area according to the method of Yuan et al. [24].

### Reverse transcription-polymerase chain reaction analysis

The mRNA expression was analyzed by reverse transcription-polymerase chain reaction (RT-PCR). Total RNA were extracted from four different cell lines (HNSC cells (HSC-6, SCC-15, SCC-25) vs normal head and neck cells (NOK)) by using TRIzol® Reagent. After identification purity and concentration, RNA was reverse transcribed to cDNA using M-MLV Reverse Transcriptase (Promega, U.S.A.). Real-time PCR amplification was performed using an SYBR Green Realtime PCR Master Mix (Toyobo, Japan). The mRNA expression level was normalized to the expression level of the housekeeping gene GAPDH. Relative gene expression levels were calculated using 2^−△△Ct^. Each experiment was performed in triplicate.

### Western blot analysis

Proteins were also extracted from the above cell lines. From sample preparation to detection, the reagents for your western blot (WB) were in one convenient kit: Western Blotting Application Solutions Kit. After sample preparation, equal amounts of sample protein were loaded onto an SDS-PAGE gel. Electrophoresis and transfer of the samples to a nitrocellulose membrane were performed. The membrane was blocked for 2 h at room temperature and incubated overnight at 4°C. Primary antibodies:anti-IGF2BP2 (1:1000, Bioworlde) and the secondary antibodies were from Southern Biotech and Boster Wuhan Biological Engineering Co., Ltd. Proteins were detected by using (Medical X-ray film, Kodak). Each experiment was performed in triplicate.

### Immune infiltration analysis in TISIDB

The correlation between the hub m6A regulator and tumor-infiltrating lymphocytes across human cancers was analyzed in TISIDB, which is a comprehensive repository portal for immune infiltration analysis (http://cis.hku.hk/TISIDB/index.php). In addition, the associations between IGF2BP2 expression and tumor grade or cancer stage in pan-cancer were also explored in TISIDB [25].

### Gene mutation analysis in cBioPortal

The genomic profile of the hub m6A regulator was analyzed by the cBioPortal (www.cbioportal.org) [26]. Mutations, putative copy-number alterations from GISTIC and mRNA Expression z-Scores (RNASeq V2 RSEM) with a z-score threshold ±1.8 were involved in it. Its association with OS of HNSC patients was displayed as Kaplan–Meier plots. The log-rank *P-*value<0.05 was considered statistically significant.

### Gene set enrichment analysis in LinkedOmics

To predict functions of the hub m6A regulators, we performed gene set enrichment analysis (GSEA) by using LinkedOmics (http://http://linkedomics.org/) [27]. Gene Ontology (GO) and Kyoto Encyclopedia of Genes and Genomes (KEGG) modules were explored in it. The minimum number of gene size was 3, and the simulation was 500. The false discovery rate (FDR) value <0.05 was statistically significant. The enrichment results were set to weighted set cover.

### Protein–protein interaction analysis in String

To predict targets of the hub m6A regulator, we performed the protein–protein interaction (PPI) network analysis by String (https://string-db.org/), which can collect, score, and integrate all publicly available sources of PPI information, and to complement these with computational predictions [28]. The related genes used therein were obtained from the above-mentioned correlation analysis.

### mRNA Expression profiling analysis in TIMER

The expression profiling of the hub m6A regulator between tumor types and adjacent normal tissues in TCGA was explored using the TIMER database (http://timer.comp-genomics.org/). [29]. The differences in transcriptional level were compared by *t-*test, and *P*<0.05 was considered statistically significant.

### Survival profiling analysis in GEPIA2

The prognostic value of the hub m6A regulator in pan-cancer and a large cancer group (including all cancer patients in TCGA) was analyzed in GEPIA2 (http://gepia2.cancer-pku.cn/#index) [30]. It was considered statistically significant when *P*-value<0.05 in GEPIA2.

## Results

### Overexpression of different m6A regulators in HNSC patients

**Figure 1:**
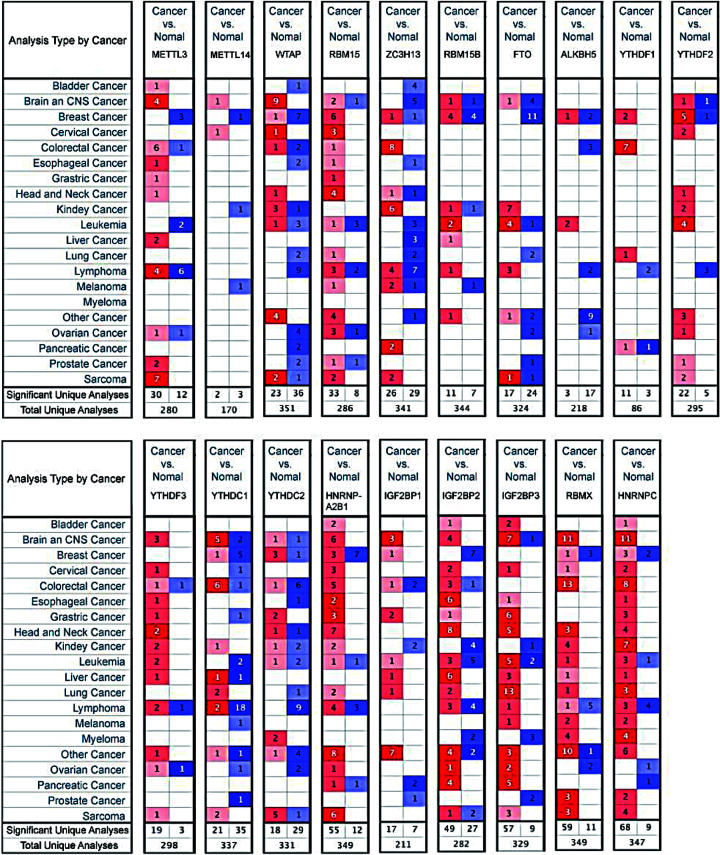
The mRNA expression of different m6A regulators in 20 different types of cancers (Oncomine) The figure showed the numbers of datasets with statistically significant mRNA overexpression (red) or downregulated expression (blue) of m6A regulators. The difference in transcriptional expression was analyzed by t test. Cut-off of p value and fold change were as follows: *P*=0.01; fold change, 1.5; gene rank, 10%; data type, mRNA.

**Figure 2:**
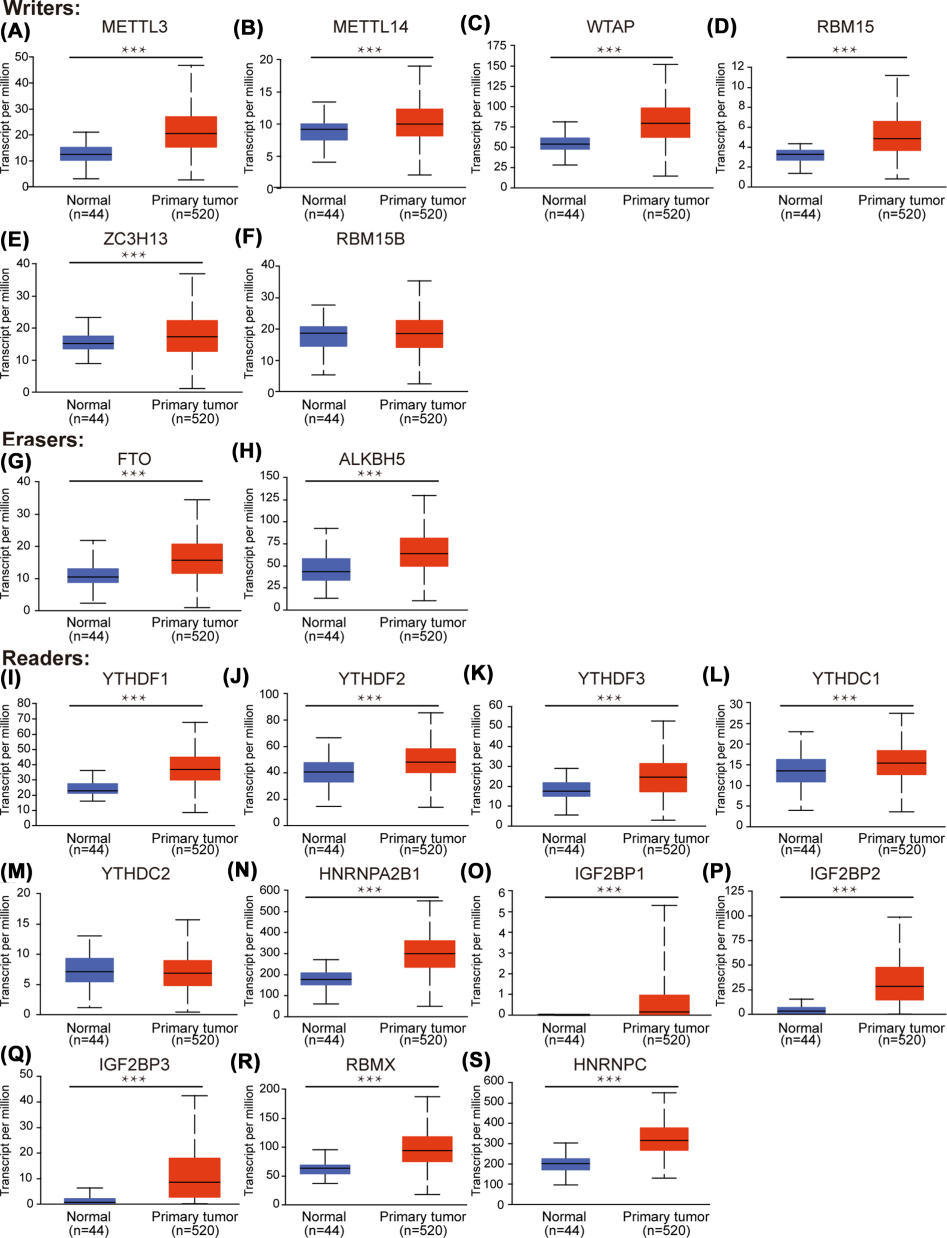
The mRNA expression of different m6A regulators in HNSC and normal controls (UALCAN) (**A**) METTL3. (**B**) METTL14. (**C**) WTAP. (**D**) RBM15. (**E**) ZC3H13. (**F**) RBM15B. (**G**) FTO. (**H**) ALKBH5. (**I**) YTHDF1. (**J**) YTHDF2. (**K**) YTHDF3. (**L**) YTHDC1. (**M**) YTHDC2. (**N**) HNRNPA2B1. (**O**) IGF2BP1. (**P**) IGF2BP2. (**Q**) IGF2BP3. (**R**) RBMX. (**S**) HNRNPC. ^***^P<0.001, ^**^P<0.01, ^*^P<0.05.

In order to explore the transcriptional expression level of different m6A regulator in HNSC, the mRNA expressions of them were analyzed in UALCAN and ONCOMINE. As shown in Figure 1, the mRNA expressions of 19 m6A regulators among over 20 types of cancers were measured in ONCOMINE. The result showed that the mRNA levels of most m6A regulators were up-regulated in cancer, especially in head and neck cancer. Then, to verify the expression differences, we used UALCAN to measure the mRNA expression of these regulators in HNSC specifically (Figure 2) (*P*<0.001). The results were consistent and almost all m6A regulators had a higher expression at mRNA level in HNSC. Therefore, we could conclude that m6A regulators showed a higher mRNA expression trend in HNSC generally, which might indicate that m6A modification is active in HNSC. In addition, since RBM15B (Figure 2F), YTHDC2 (Figure 2M) and ZC3H13 (Figure 1) did not have positive results in analyses, we excluded them in the following analyses.

### The prognostic value of the selected m6A regulators in patients with HNSC

**Figure 3:**
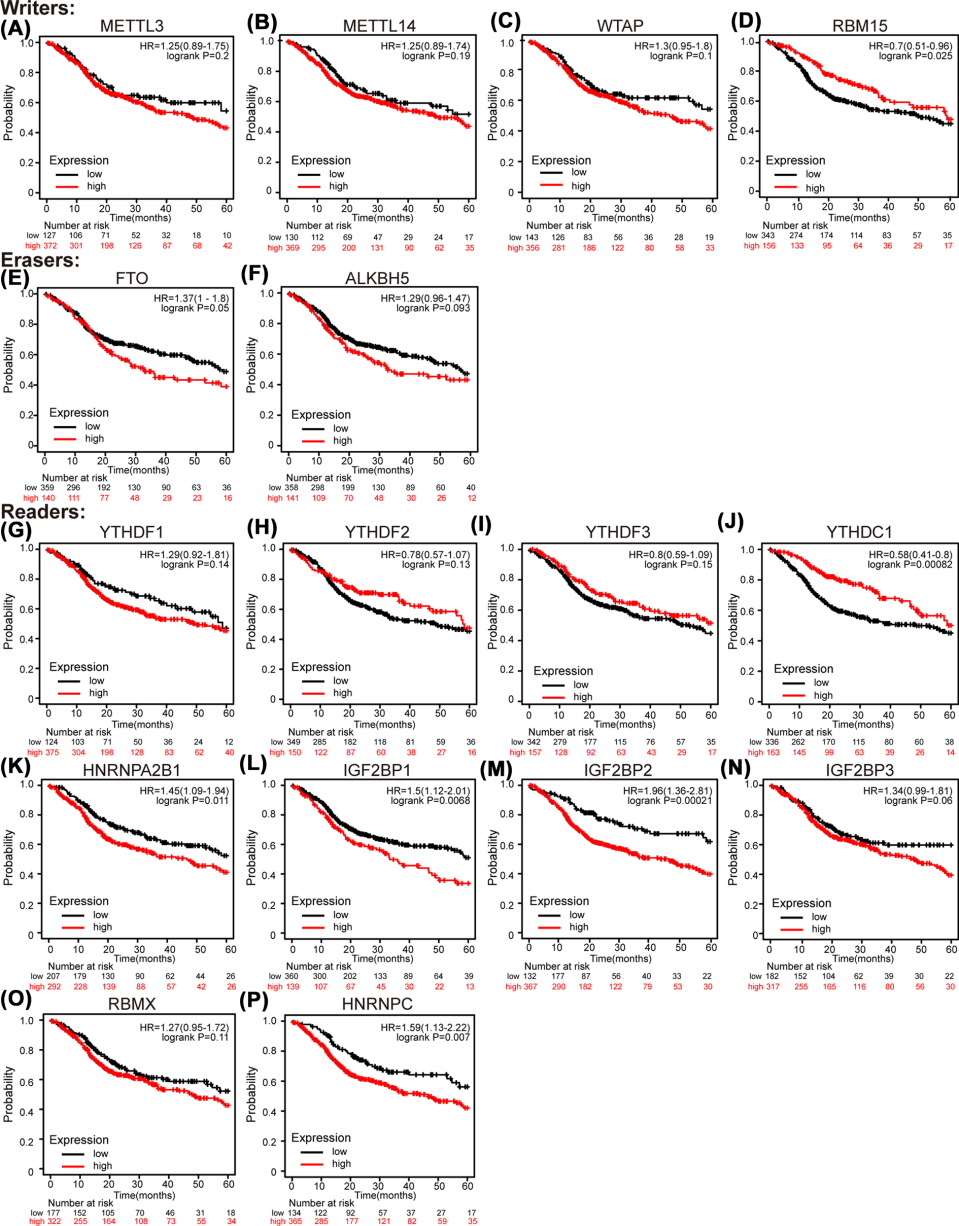
The survival plot of the filtered m6A regulators in HNSC patients (Kaplan-Meier Plotter) (**A**) METTL3. (**B**) METTL14. (**C**) WTAP. (**D**) RBM15. (**E**) FTO. (**F**) ALKBH5. (**G**) YTHDF1. (**H**). YTHDF2. (**I**) YTHDF3. (**J**) YTHDC1. (**K**) HNRNPA2B1. (**L**) IGF2BP1. (**M**) IGF2BP2. (**N**) IGF2BP3. (**O**) RBMX. (**S**) HNRNPC.

To further select m6A regulators that correlate to the prognosis of HNSC patients, we utilized Kaplan–Meier plotter to study the relationship between expression levels of the selected m6A regulatory genes and the survival of HNSC patients. As shown in Figure 3, the higher mRNA expressions of HNRNPA2B1 (hazard ratio (HR) = 1.45, 95% Confidence Interval (CI): 1.09–1.94, and *P*=0.011) (Figure 3K), IGF2BP1 (HR = 1.5, 95% CI: 1.12–2.01, and *P*=0.0068) (Figure 3L), IGF2BP2 (HR = 1.96, 95% CI: 1.36–2.81, and *P*=0.00021) (Figure 3M), and HNRNPC (HR = 1.59, 95% CI: 1.13–2.22, and *P*=0.007) (Figure 3P) were significantly associated with shorter 5-years OS of HNSC patients, while the higher mRNA expressions of RBM15 (HR = 0.70, 95% CI: 0.51–0.96, and *P*=0.025) (Figure 3D) and YTHDC1 (HR = 0.58, 95% CI: 0.41–0.8, and *P*=0.00082) (Figure 3J) were significantly associated with favorable outcome of HNSC patients. Then, to validate the results, the univariate and multivariate Cox regression analyses were conducted based on data of 502 HNSC patients from TCGA. As shown in Table 1, higher M stage, lymphovascular invasion, and IGF2BP2 expression were significantly associated with poor OS of patients with HNSC in the univariate analysis. The multivariate analysis showed that M stage (HR = 4.821, 95% CI: 1.144–20.317, and *P*=0.032), lymphovascular invasion (HR = 1.681, 95% CI: 1.167–2.420, and *P*=0.005), and IGF2BP2 (HR = 1.458, 95% CI: 1.013–2.098, and *P*=0.042) were independent prognostic factors of unfavorable OS for patients with HNSC, which indicated that IGF2BP2 might be the most significant m6A regulator.

**Table 1:** The univariate and multivariate Cox regression analyses of OS and clinicopathologic parameters in HNSC patients

Characteristics	Total (N)	Univariate analysis		Multivariate analysis	
		Hazard ratio (95% CI)	*P*-value	Hazard ratio (95% CI)	*P*-value
T stage	484				
T1 & T2	175	Reference			
T3 & T4	309	1.230 (0.921–1.642)	0.160		
N stage	477				
N0	238	Reference			
N1 & N2 & N3	239	1.257 (0.960–1.647)	0.097	1.218 (0.846–1.753)	0.288
M stage	474				
M0	469	Reference			
M1	5	4.794 (1.765–13.016)	**0.002**	4.821 (1.144–20.317)	**0.032**
Clinical stage	485				
Stage I & Stage II	113	Reference			
Stage III & Stage IV	372	1.214 (0.875–1.683)	0.245		
Gender	499				
Female	133	Reference			
Male	366	0.754 (0.566–1.004)	0.054	0.761 (0.515–1.126)	0.172
Age	499				
<=60	244	Reference			
>60	255	1.238 (0.945–1.621)	0.122		
Histologic grade	480				
G1 & G2	359	Reference			
G3 & G4	121	0.942 (0.690-1.287)	0.709		
Lymphovascular invasion	338	–			
No	218	Reference			
Yes	120	1.688 (1.201–2.371)	**0.003**	1.681 (1.167–2.420)	**0.005**
IGF2BP1	499				
Low	249	Reference			
High	250	1.095 (0.837-1.433)	0.506		
IGF2BP2	499	–			
Low	249	Reference			
High	250	1.431 (1.092–1.875)	**0.009**	1.458 (1.013–2.098)	**0.042**
YTHDC1	499				
Low	249	Reference			
High	250	0.853 (0.652–1.116)	0.247		
HNRNPA2B1	499				
Low	249	Reference			
High	250	1.240 (0.948–1.621)	0.116		
RBM15	499				
Low	249	Reference			
High	250	0.831 (0.636–1.085)	0.174		
HNRNPC	499				
Low	249	Reference			
High	250	1.198 (0.915–1.568)	0.189		

*P*<0.05 was considered statistically significant in all analyses and highlighted in bold.

### Validation of the overexpression of IGF2BP2 in HNSC

**Figure 4:**
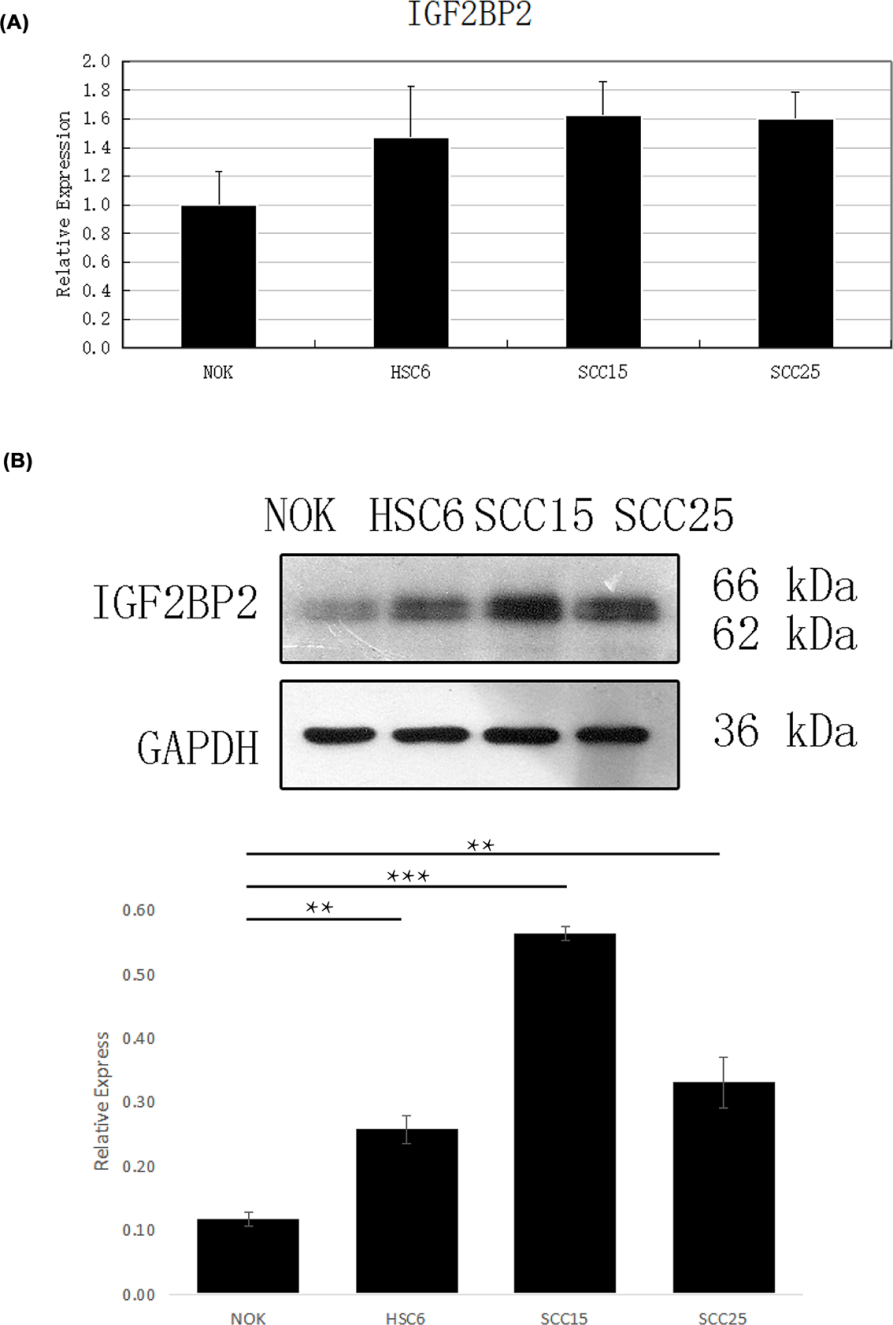
The expression level of IGF2BP6 in vitro (**A**) IGF2BP2 mRNA levels in HNSC cells and normal head and neck cells. (**B**) IGF2BP2 protein expression in HNSC cells and normal head and neck cells. ^***^P<0.001, ^**^P<0.01.

**Figure 5:**
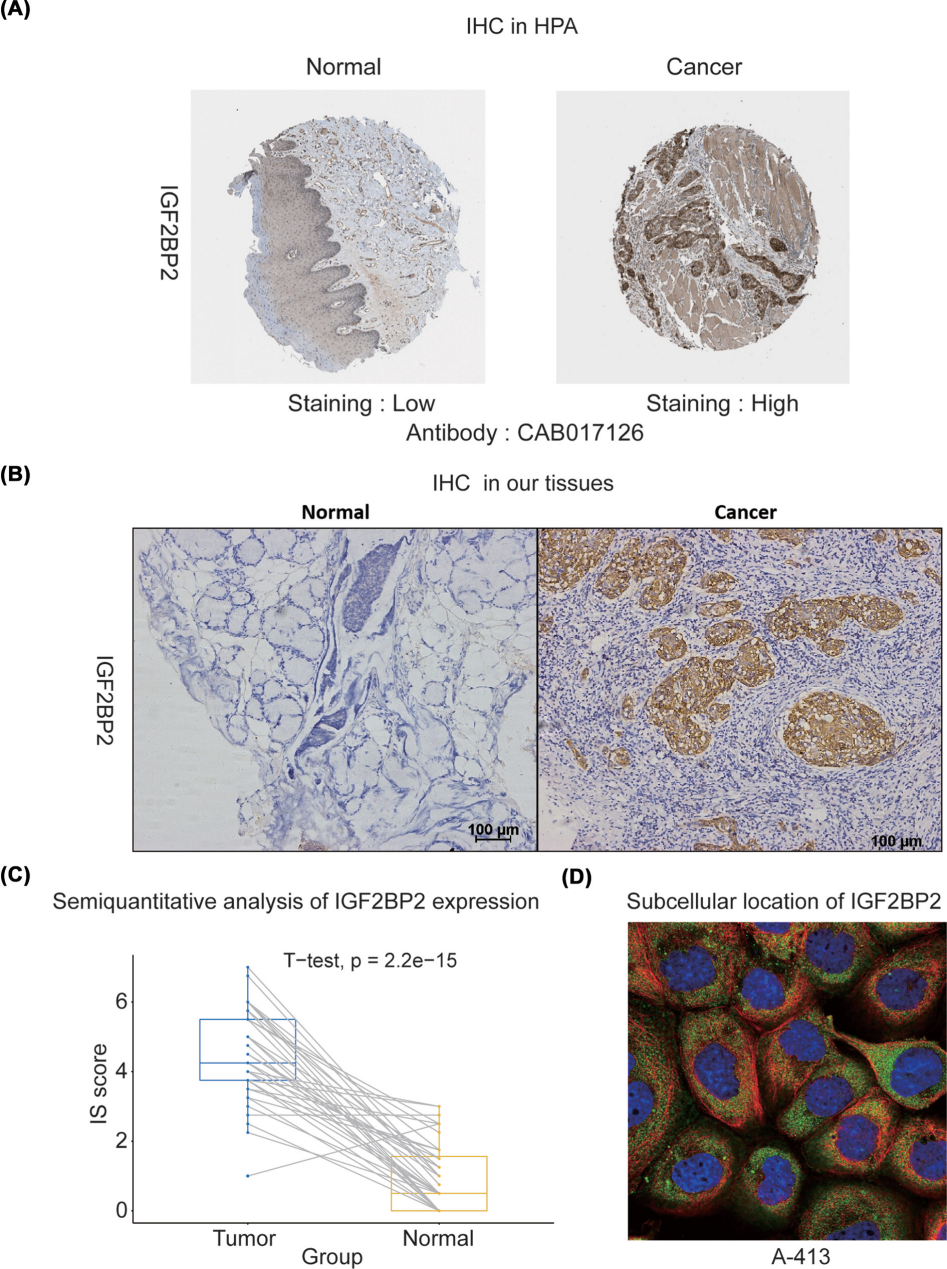
The protein expression of IGF2BP2 in HNSC patients in HPA and our own dataset (**A**) The protein expression of IGF2BP2 in HPA. (**B**) The protein expression of IGF2BP2 in our own dataset. (**C**) Semiquantitative analysis of IGF2BP2 protein expression in IHC based on our own dataset. (**D**) The subcellular location of IGF2BP2 in A-413 cell line. The image shows the markers for the IGF2BP2 proteins (green), nucleus (blue), microtubules (red) (HPA).

In order to verify the higher expression of IGF2BP2 in HNSC when compared with normal tissue, we used four cell lines (three HNSC cell lines (HSC-6, SCC-15, SCC-25) vs one normal control cell line (NOK) to explore the mRNA and protein expression by QRT-PCR and WB. As shown in Figure 4, the mRNA levels in HNSC cells were higher than in normal control cells. The WB assay result found that the protein levels of these HNSC cells were also significantly higher than in normal control cells (*P*<0.01). Then, the protein levels of IGF2BP2 were further explored in HPA and our own clinical samples (*n*=40 pairs) based on IHC assay. As shown in Figure 5, the protein expression of IGF2BP2 was up-regulated in HNSC compared with normal controls in HPA. In our samples, the protein level of IGF2BP2 was also significantly up-regulated in HNSC samples compared with the paired normal tissues (mean IS = 4.43 vs 0.98, *P*<0.001). In addition, the subcellular location of IGF2BP2 localized mainly to the cytosol in three different cell lines (A-431, U-2 OS, U-251 MG) in HPA (Figure 5D, Supplementary Figure S1A,B), which indicated that IGF2BP2 mainly makes function in cytosol [23].

### The correlation between IGF2BP2 and clinical factors in HNSC

**Figure 6:**
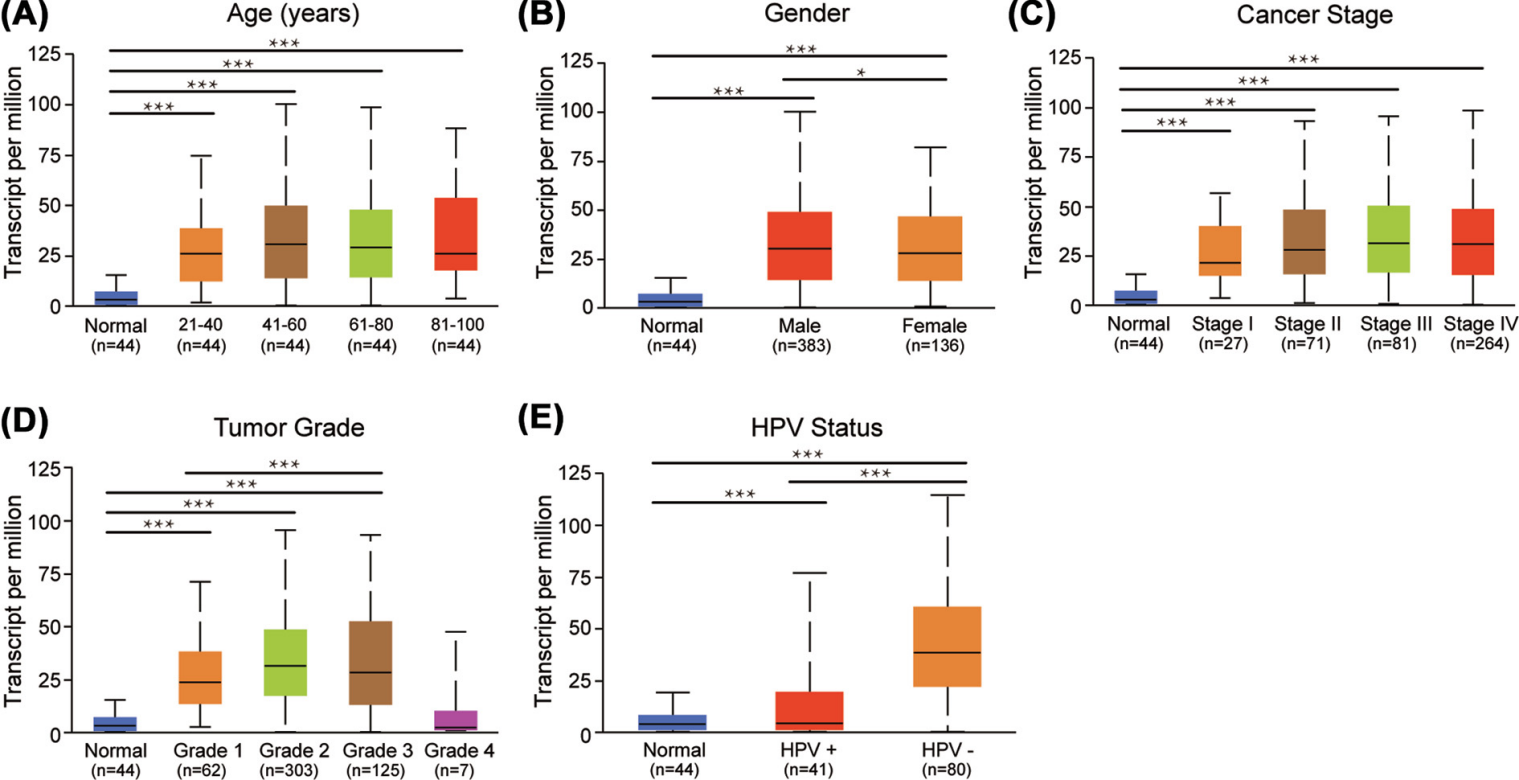
The mRNA expression of IGF2BP2 in different clinical features (UALCAN) (**A**) Age. (**B**) Gender. (**C**) Cancer Stage. (**D**) Tumor Grade. (**E**) HPA status. ^***^P< 0.001, **P<0.01, ^*^P<0.05.

Then, we used UALCAN to further explore the relationship between the mRNA expression of IGF2BP2 and clinicopathological parameters in HNSC patients (age, gender, cancer stages, tumor grades, HPV status). As shown in Figure 6, the mRNA expression of IGF2BP2 was only significantly associated with HPV status (*P*<0.001). The higher mRNA expression of IGF2BP2 was found in HPV- HNSC patients compared with HPV+ HNSC patients.

### The correlation between IGF2BP2 and tumor immune infiltration

**Figure 7:**
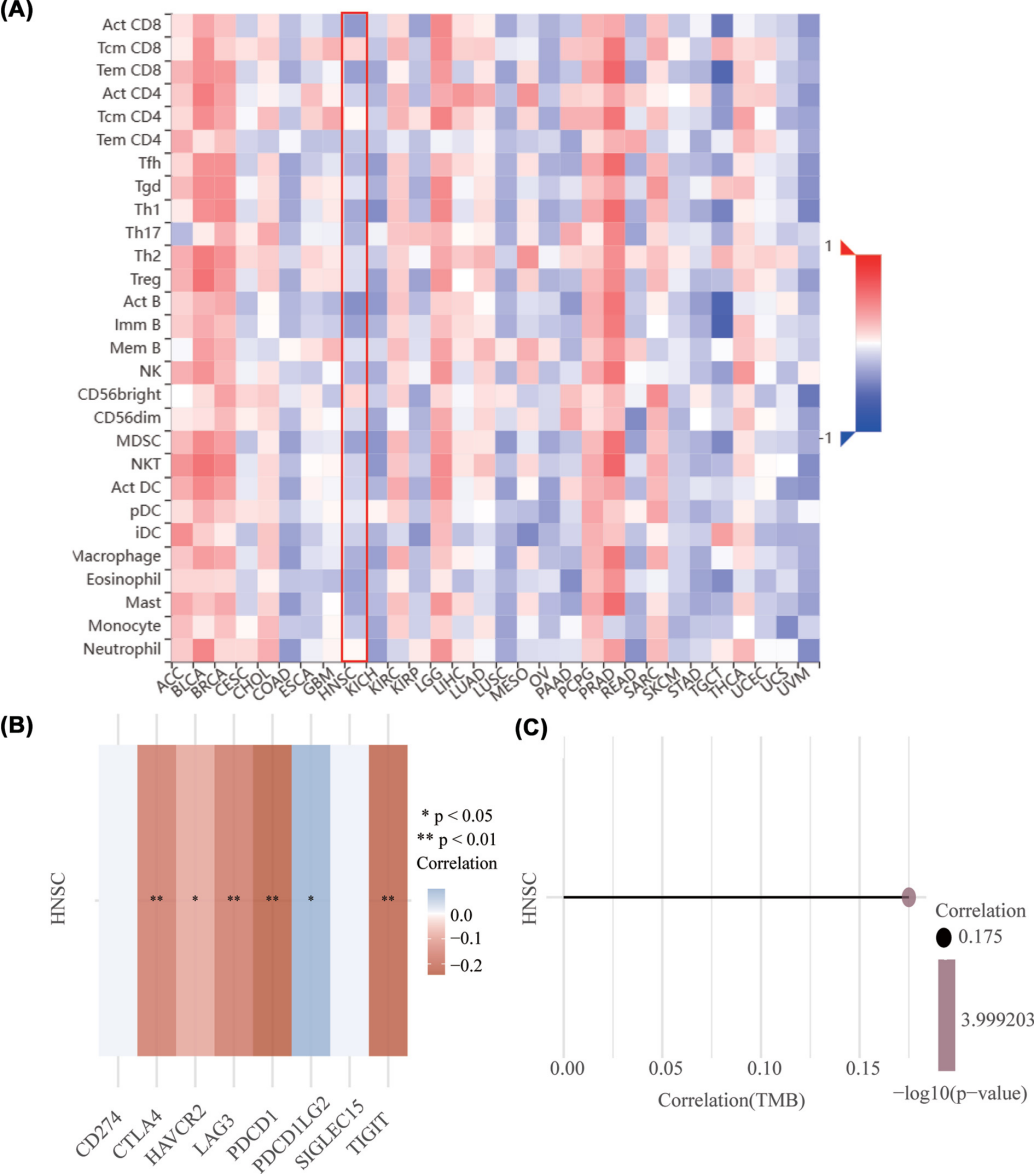
The relationship between IGF2BP2 expression and Immunity (**A**) Relations between abundance of tumor-infiltrating lymphocytes (TILs) and IGF2BP2 (TSIDB). (**B**) The relationship between IGF2BP2 expression immune-checkpoint-related gene expression. ^***^P<0.001, ^**^P<0.01, ^*^P<0.05. Different colors represent the changes of correlation coefficients. (**C**) Spearman correlation analysis of tumor mutation burden (TMB) and IGF2BP2 gene expression. The size of the dots represents the size of the correlation coefficient, and different colors represent the significance of p value. The bluer the color, the smaller the p value.

Tumor immune infiltration plays an important role in HNSC progression. Therefore, we explored whether IGF2BP2 could influence immune infiltration by using TISIDB. As shown in Figure 7A, the expression of IGF2BP2 was negatively correlated with 28 tumor-infiltrating lymphocyte (TIL) types generally in TISIDB. In immune checkpoint correlation analysis, the expression of IGF2BP2 was significantly negatively correlated with most immune checkpoint-related genes, such as CTLA4, HAVCR2, LAG3, PDCD1, TIGIT (Figure 7B). In addition, the expression of IGF2BP2 was positively correlated with TMB (Figure 7C). In conclusion, these results indicated that IGF2BP2 expression might lead to poor prognosis of HNSC patients by suppressing tumor immune infiltration.

### Genetic mutations in IGF2BP2 and their associations with OS in HNSC

**Figure 8:**
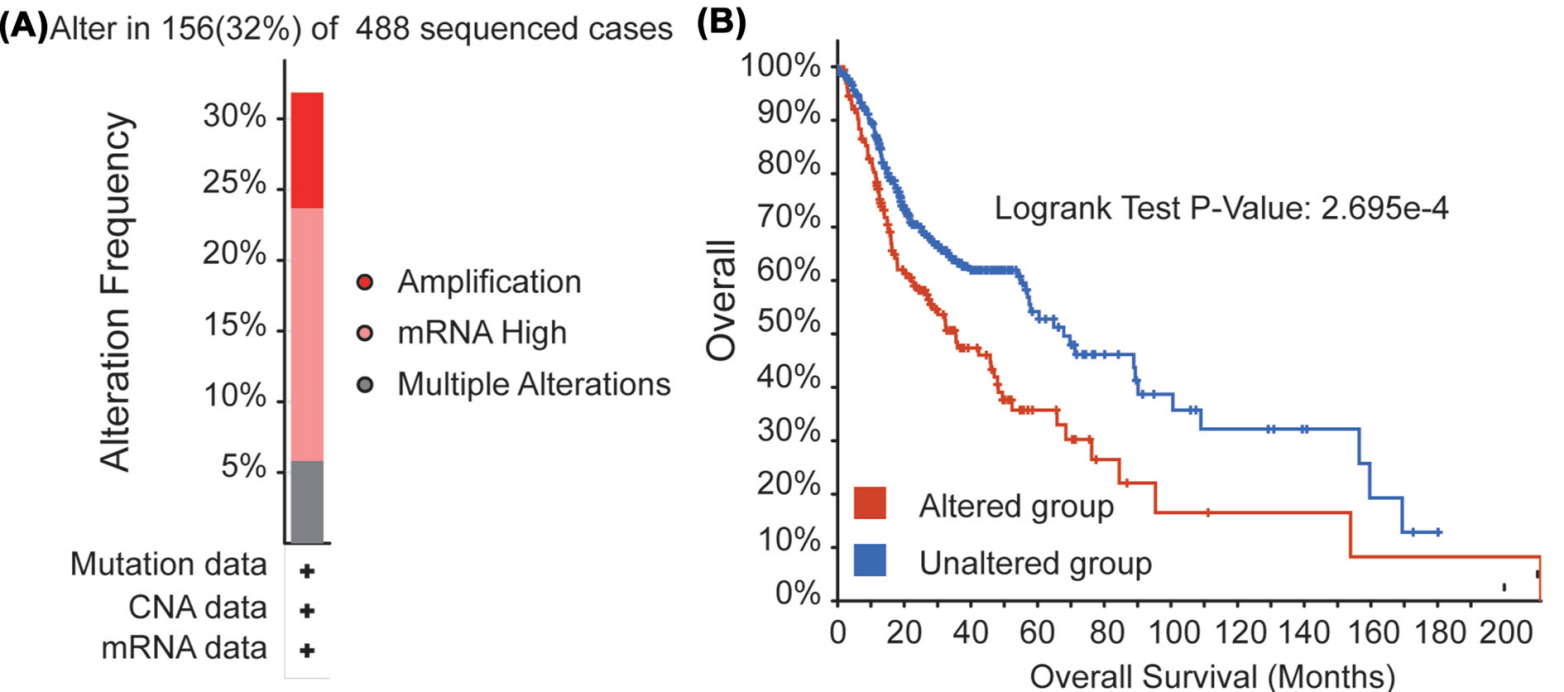
Genetic mutations in IGF2BP2 and their association with survival in HNSC (cBioPortal) (**A**) Gene mutation types and frequency of IGF2BP2 in HNSC patients. (**B**) The survival plot of genetic mutations in HNSC.

Next, we analyzed genetic alterations of IGF2BP2 in HNSC and their associations with OS by using cBioPortal. As shown in Figure 8A, among the 488 sequenced cases, 156 cases of genetic alterations were found, with a mutation rate of 32%. The main types of alterations are mRNA high and amplification. Furthermore, the result of Kaplan–Meier plot and log-rank test showed that genetic alterations in IGF2BP2 was associated with shorter OS (Figure 8B, *P*<0.001) of HNSC patients. Since these two mutation types were closely related to high expression of IGF2BP2, it also indicated that the high expression of IGF2BP2 was associated with the poor prognosis of HNSC patients.

### GSEA analysis of IGF2BP2

**Figure 9:**
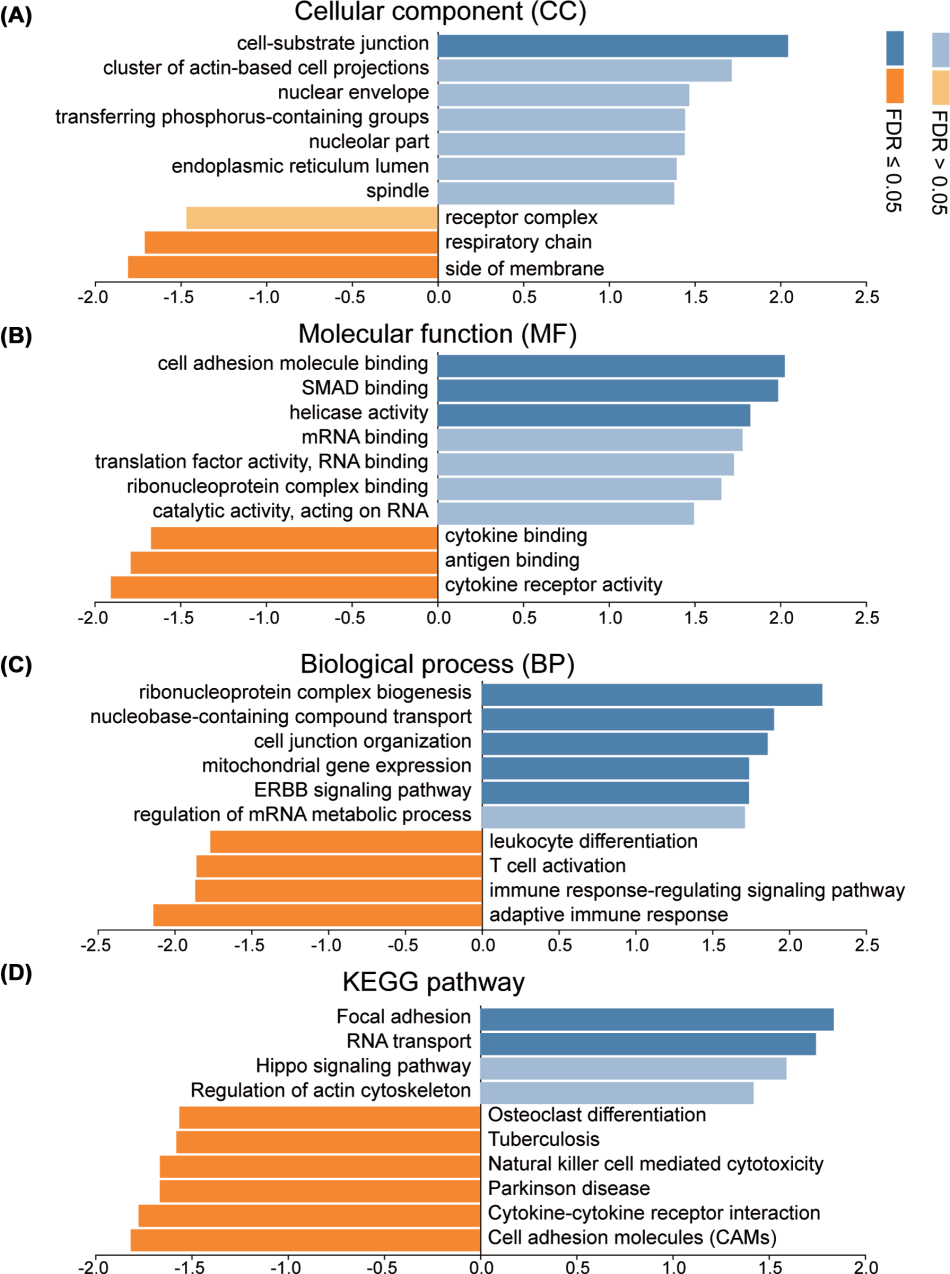
The functional analysis of IGF2BP2 in HNSC (LinkedOmics) (**A**) GO: cellular component. (**B**) GO: molecular function. (**C**) GO: biological process (**D**) KEGG pathway. False discovery rate (FDR).

To predict the function of IGF2BP2, we then used LinkedOmics to perform GO and KEGG analyses through the GSEA module. From the results, the high expression of IGF2BP2 was associated with cell-substrate junction in cellular component (CC) module, while the low expression of IGF2BP2 was associated with side of membrane and respiratory chain (Figure 9A). In molecular function (MF) module, the high expression of IGF2BP2 was related to cell adhesion molecule binding, SMAD binding and helicase activity, and the low expression of it was related to cytokine receptor activity, antigen-binding and cytokine-binding (Figure 9B). As for biological process (BP), its high expression was associated with ribonucleoprotein complex biogenesis, nucleobase-containing compound transport, etc., and the low expression of it was associated with adaptive immune response, immune response-regulating signaling pathway, T-cell activation, etc. (Figure 9C). In KEGG pathway, the high expression of it was related to focal adhesion and RNA transport, while the low expression of it was related to cell adhesion molecules (CAMs), cytokine–cytokine receptor interaction, natural killer cell-mediated cytotoxicity, etc. (Figure 9D). It was worth noting that the functional analysis also proved IGF2BP2’s relationship with immunity.

### The PPI network analysis based on IGF2BP2

**Figure 10:**
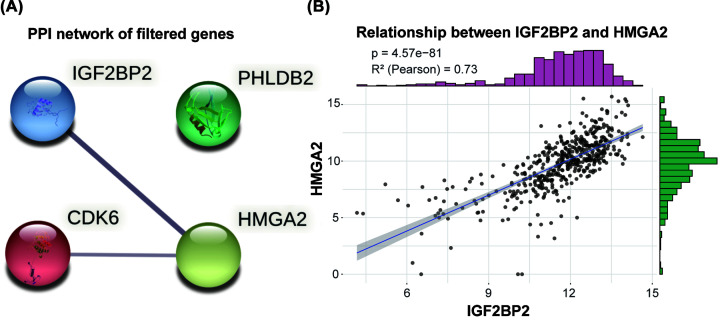
The PPI network analysis based on IGF2BP2 and its correlated genes (**A**) The PPI network of IGF2BP2 and its co-expression genes (String). (**B**) The expression pattern between IGF2BP2 and HMGA2.

To explore the potential targets of IGF2BP2, we conducted the PPI network analysis by using String. After performing correlation analysis between IGF2BP2 and all genes in HNSC, we finally got three representatively filtered genes (CD6, HMGA2, PHLDB2) that were significantly correlated with IGF2BP2 expression (*r* > 0.6). In String, only HMGA2 was directly associated with IGF2BP2 (Figure 10A). Its expression pattern was similar to IGF2BP2 expression (*r* = 0.73) in the correlation analysis (Figure 10B).

### Generalization value of IGF2BP2 in pan-cancer

**Figure 11:**
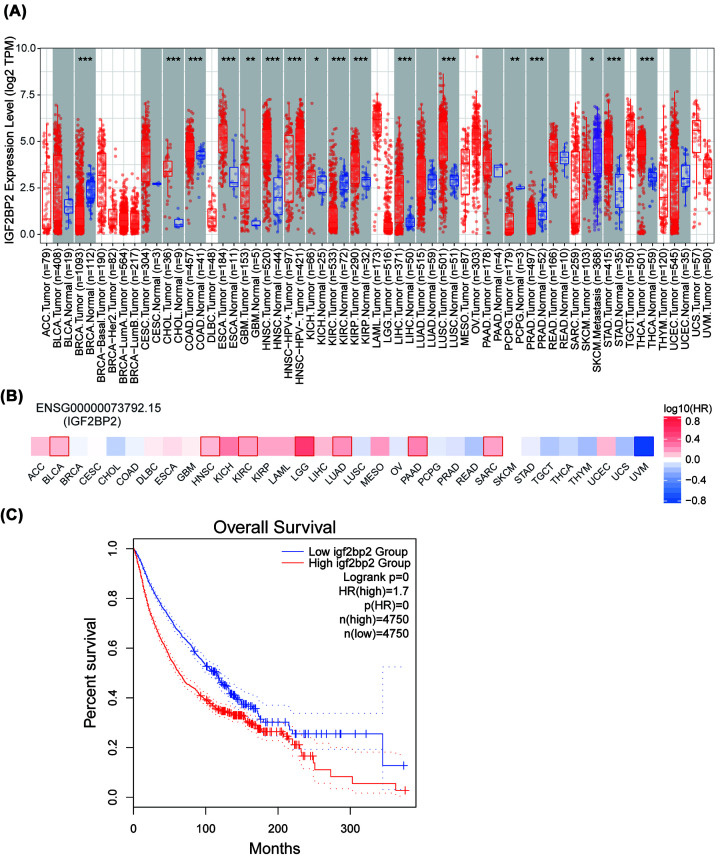
Generalization value of IGF2BP2 in pan-cancer (**A**) Comparison of AHNAK2 mRNA expression between pan-cancer cancerous and para-cancerous tissues (TIMER). ^***^P<0.001, ^**^P<0.01, ^*^P<0.05. (**B**) Associations between IGF2BP2 expression and overall survival across human cancers. Boxes with bold edge represent statistical significance in difference. Different colors represent different size of log10 hazard ration (HR) value (GEPIA2). (**C**) K-M survival analysis of pan-cancer in different IGF2BP2 groups (GEPIA2).

To investigate whether IGF2BP2 has broad value, we conducted further studies on IGF2BP2 across all cancers. Oncomine and TIMER showed that IGF2BP2 expression status varies in different cancers and most cancer types present higher IGF2BP2 expression (Figures 2 and 11A). TISIDB showed that the high expression of IGF2BP2 in pan-cancer tended to be accompanied by a higher tumor grade and cancer stage (Supplementary Figure S2A,B). In GEPIA2, we found the higher IGF2BP2 expression was significantly associated with poor prognosis of most cancers, such as BLCA, GBM, HNSC, KIRC, LGG, LUAD, PAAD, SARC (Figure 11B). In a large number of pan-cancer samples (*n*=9500), the higher IGF2BP2 was significantly correlated with poorer survival of cancer patients (HR = 1.7) (Figure 11C).

## Discussion

M6A methylation plays a significant role in tumorigenesis and progression [8,9]. The modification is reversible and dynamic, which is regulated by specific types of m6A regulators (writer, eraser, reader) [8–10]. Although m6A is of great value in cancers, the relationship between m6A regulators and HNSC has largely remained unexplored. Among them, METTL3 is studied the most and plays an unfavorable role in HNSC. METTL3 could stabilize the mRNA expression of target genes in studies. It was reported that METTL3 promoted tumorigenesis and metastasis through BMI1 m6A methylation [18]. Another study reported that METTL3-mediated m6A modification played an oncogenic role by regulating DNp63 [31]. However, besides METTL3, there are still a large number of m6A regulators in HNSC that have not been studied. It is costly to perform wet-lab experiments to study them one by one. Therefore, we utilized bioinformatics methods to study the clinical significance of all m6A regulators in HNSC and aim to find out the hub regulators, which might offer direction for future research and improve the prognosis of HNSC patients.

In our study, we found overall overexpression of m6A regulator in HNSC. Most importantly, IGF2BP2 was found to be the hub m6A regulator among all regulators, which was an independently negative prognostic factor in HNSC. Compared with the normal controls, it was up-regulated at the mRNA and protein levels in HNSC. The higher expression of IGF2BP2 was associated with poor prognosis. To figure out the functions of IGF2BP2, we conducted further analyses on it. In the stratified analysis, the higher expression level of IGF2BP2 mRNA was found in HPV patients with HNSC. It indicated that IGF2BP2 was involved in the pathogenesis of HPV-negative HNSC. However, the mechanism behind it remains uncovered. From the functional analysis, it showed that IGF2BP2 was negatively associated with immune activity. In terms of tumor immune infiltration, IGF2BP2 was found also negatively correlated with immune infiltration. In addition, the high genetic alteration rate (32%) was found in HNSC patients. The alteration types were mainly mRNA high or amplification, which represented the high expression of IGF2BP2. And genetic alteration in IGF2BP2 was significantly associated with shorter OS. Furthermore, IGF2BP2 was predicted to interact with HMGA2 in the PPI analysis. HMGA2 mRNA expression was positively correlated with the expression of IGF2BP2. HMGA2 protein contains structural DNA-binding domains and may act as a transcriptional regulating factor. According to the literature, HMGA2 was an upstream regulator of IGF2BP2. HMGA2 could bind to a proximal region of IGF2BP2 and activate its transcription [32]. In leiomyomas, HMGA2 overexpression up-regulated IGF2BP2 and pAKT, which played an important role in the IGF2BP2-mediated pAKT activity in angiogenesis [33]. Finally, in pan-cancer analysis, IGF2BP2 was up-regulated in most cancers and its overexpression was associated with poorer prognosis of cancer patients.

So far, there are few studies studying on IGF2BP2 in HNSC. Recently, there were studies found that the expression of IGF2BP2 was up-regulated in both gene and protein levels [34,35]. Yu et al. knockdown and overexpressed IGF2BP2 in FaDu and SCC15 cells and found that overexpression of IGF2BP2 promotes the migration and invasiveness of HNSC cells by wound-healing analysis and transwell analysis. Mechanically, they found that IGF2BP2 could stabilize slug mRNA via m6A, and then promoted lymphatic metastasis and epithelialmesenchymal transition in HNSC [34]. In addition, previous study also found that IGF2BP2 polymorphisms were associated with less favorable oral cancer clinical characteristics [36]. Our study predicted many potential functions of IGF2BP2 in HNSC, especially with immune function. In other cancer types, IGF2BP2 had also been found to play a carcinogenic effect. In pancreatic cancer, it was activated by genomic amplification and miR-141 silencing [37]. Then, IGF2BP2 was highly expressed in thyroid cancer, and MALAT1 was found to up-regulate IGF2BP2 and enhance MYC expression via m6A modification recognition by competitively binding to miR-204 [38]. Our study showed a general unfavorable role of IGF2BP2 in cancers, which might have a broad application among cancers and deserves further study.

Some limitations also existed in our study. First, we only proved that IGF2BP2 had prognostic value in HNSC in TCGA dataset, but there was no other follow-up data to verify the result. Therefore, a prospective study consisting of large samples is needed in future research. Second, although we predict the underlying mechanism of IGF2BP2, we did not conduct the mechanism studies to validate IGF2BP2’s m6A role. And the m6A mechanism between IGF2PB2 and hub genes (such as HMGA2) was not verified with experimental data. Further studies will be performed to prove our results. Finally, we did not explore the diagnostic and therapeutic roles of IGF2BP2. It was worth studying the possibilities of IGF2BP2 as a diagnostic or therapeutic biomarker in the future.

In conclusion, our results indicated that general overexpression of m6A regulators was found in HNSC when compared with normal controls. Among them, IGF2BP2 was considered to be the most significant m6A regulator. Its expression predicted the poor prognosis of HNSC patients independently. mRNA high or amplification were mainly mutation types of HNSC patients (32%) in genetic mutation analysis, and it was associated with shorter OS. In addition, IGF2BP2 might be closely related to immune function, especially negatively related to immune cells infiltration. HMGA2 might interact with IGF2BP2 in HNSC. From these results, IGF2BP2 might serve as a core m6A regulator in HNSC, which might help to improve the prognosis of HNSC patients.

## Supplementary Material

Supplementary Figures S1-S2 and Table S1

## Data Availability

Publicly available datasets were analyzed in this study. These data can be found here: https://www.cancer.gov/about-nci/organization/ccg/research/structural-genomics/tcga, https://xenabrowser.net/datapages/.

## Abbreviations

AML: acute myeloid leukemia
BP: biological process
CAM: cell adhesion molecule
CC: cellular component
CI: confidence interval
CRC: colorectal cancer
FDR: false discovery rate
GBM: glioblastoma multiforme
GO: gene ontology
GSEA: gene set enrichment analysis
HNSC: head and neck squamous cell carcinoma
HPA: Human Protein Atlas
HPV: human papillomavirus
HR: hazard ratio
IHC: immunohistochemistry
IS: immunostaining score
KEGG: Kyoto Encyclopedia of Genes and Genomes
m6A: methylation of N6 adenosine
MF: molecular function
OS: overall survival
PPI: protein–protein interaction
RT-PCR: reverse transcription-polymerase chain reaction
TCGA: The Cancer Genome Atlas
TIL: tumor-infiltrating lymphocyte
TMB: tumor mutational burden
WB: western blot
