# Supplementary material for: IGF2BP2 serves as a core m6A regulator in head and neck squamous cell carcinoma
Source: Biosci Rep. 2022 Nov 11;42(11):BSR20221311. doi: 10.1042/BSR20221311 (PMC9653096; doi:10.1042/BSR20221311)
Supplement: Supplementary Figures S1-S2 and Table S1 [file BSR-2022-1311_supp.pdf]

A

Subcellular location of IGF2BP2

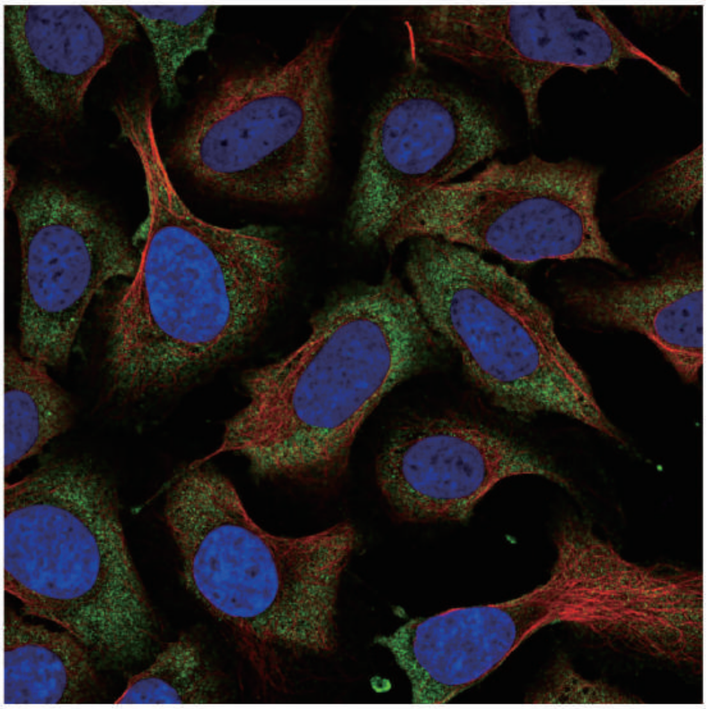

U-2 OS

B

Subcellular location of IGF2BP2

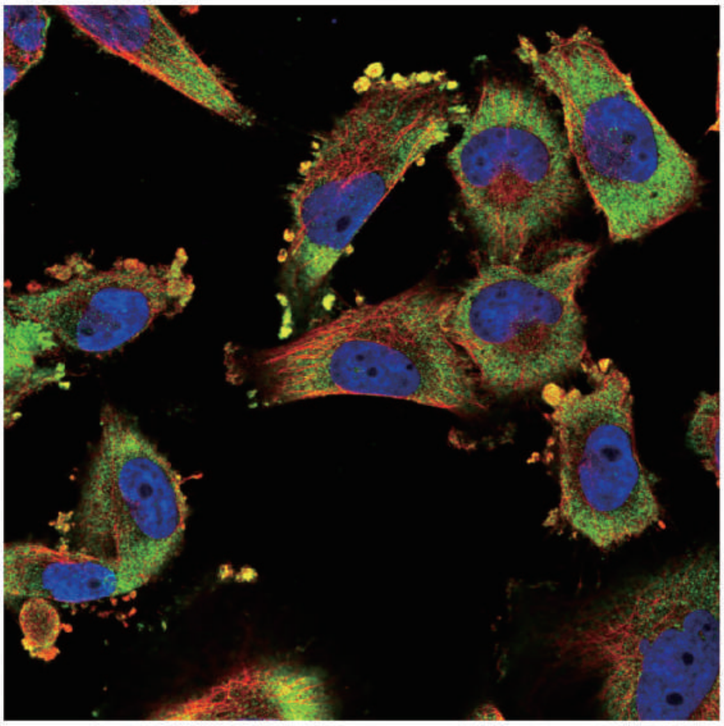

U-251 MG

Figure S1. The subcellular location of IGF2BP2 in different cell lines.
(**A**) U-2 OS. (**B**) U-251. The image shows the markers for the IGF2BP2 proteins (green), nucleus (blue), microtubules (red) (HPA).

A

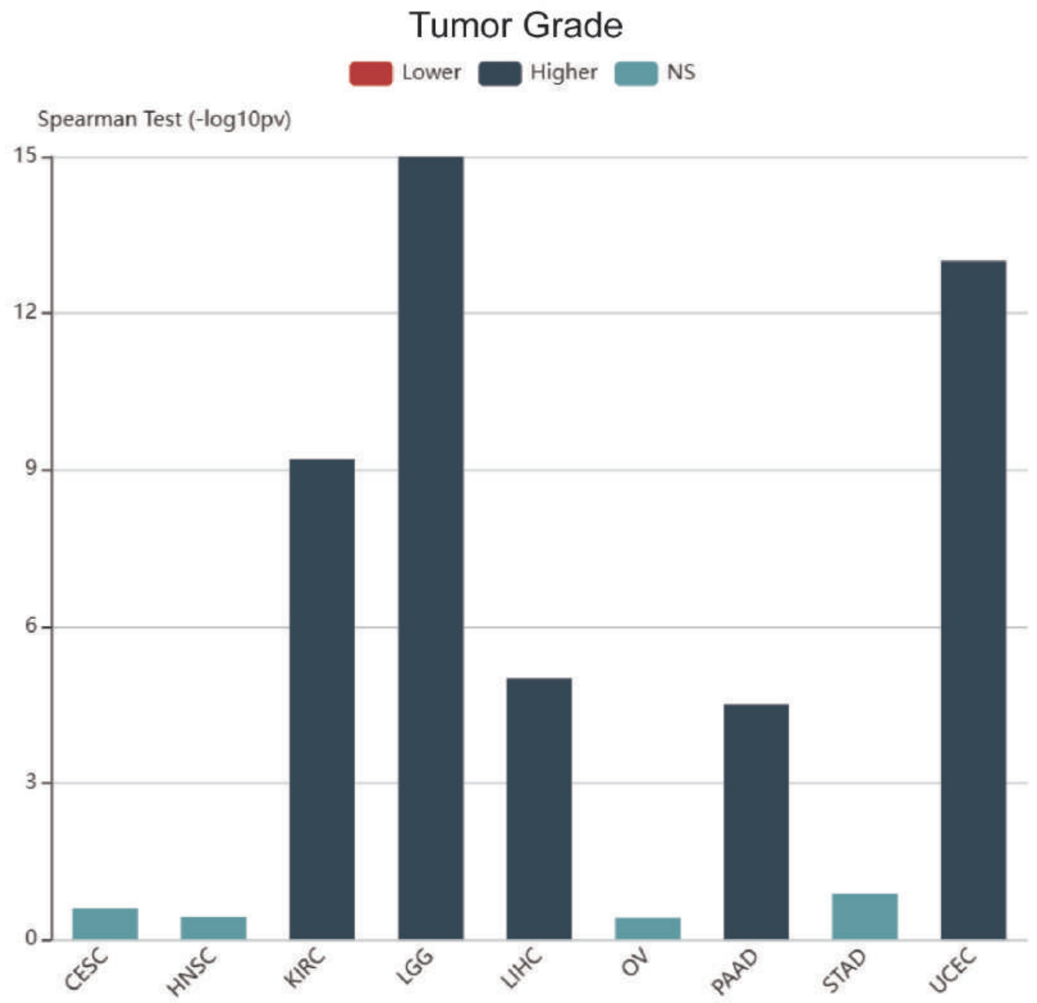

B

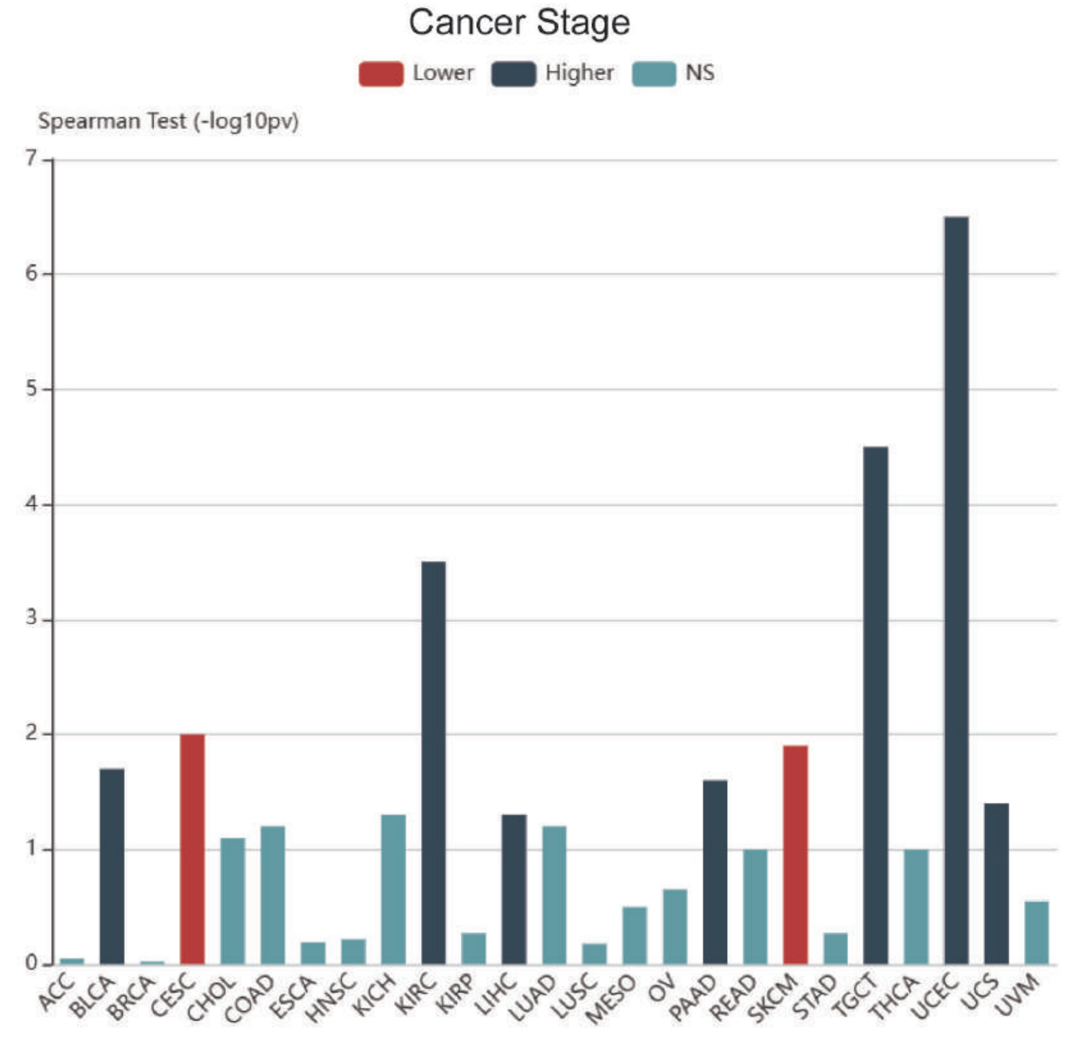

Figure S2. Associations between IGF2BP2 expression and clinical parameters across human cancers.
(**A**) tumor grade. (**B**) Cancer stage.

Table S1. The clinical baseline data of HNSC patients from TCGA (n=502).

Characteristic	levels	Overall
n		502
Age, n (%)	<=60	245 (48.9%)
	>60	256 (51.1%)
Gender, n (%)	Female	134 (26.7%)
	Male	368 (73.3%)
Race, n (%)	Asian	10 (2.1%)
	Black or African American	47 (9.7%)
	White	428 (88.2%)
T stage, n (%)	T1	33 (6.8%)
	T2	144 (29.6%)
	T3	131 (26.9%)
	T4	179 (36.8%)
N stage, n (%)	N0	239 (49.8%)
	N1	80 (16.7%)
	N2	154 (32.1%)
	N3	7 (1.5%)
M stage, n (%)	M0	472 (99%)
	M1	5 (1%)
Clinical stage, n (%)	Stage I	19 (3.9%)
	Stage II	95 (19.5%)
	Stage III	102 (20.9%)
	Stage IV	272 (55.7%)
Histologic grade, n (%)	G1	62 (12.8%)
	G2	300 (62.1%)
	G3	119 (24.6%)
	G4	2 (0.4%)
Smoker, n (%)	No	111 (22.6%)
	Yes	381 (77.4%)
Alcohol history, n (%)	No	158 (32.2%)
	Yes	333 (67.8%)
Lymphovascular invasion, n (%)	No	219 (64.2%)
	Yes	122 (35.8%)
